# Metabolomics of Breast Milk: The Importance of Phenotypes

**DOI:** 10.3390/metabo8040079

**Published:** 2018-11-20

**Authors:** Angelica Dessì, Despina Briana, Sara Corbu, Stavroula Gavrili, Flaminia Cesare Marincola, Sofia Georgantzi, Roberta Pintus, Vassilios Fanos, Ariadne Malamitsi-Puchner

**Affiliations:** 1 Neonatal Intensive Care Unit, Neonatal Pathology and Neonatal Section, Azienda University Polyclinic, University of Cagliari, 09124 Cagliari, Italy; sari.crb@gmail.com (S.C.); gomberta@icloud.com (R.P.); vafanos@tin.it (V.F.); 2 National and Kapodistrian University of Athens, 10679 Athens, Greece; brianadespina@yahoo.com (D.B.); amalpu@med.uoa.gr (A.M.-P.); 3 Neonatal Intensive Care Unit, General District, Hospital Alexandra, 11528 Athens, Greece; vpgavrili@gmail.com (S.G.); sofiageorgantzi@yahoo.gr (S.G.); 4 Department of Chemical and Geological Sciences, University of Cagliari, 09124 Cagliari, Italy; flaminia@unica.it

**Keywords:** metabolomics, human milk, NMR

## Abstract

Breast milk is the gold standard of nutrition for newborns. Its composition is tailored to the nutritional needs of the infant and varies between mothers. In recent years, several bioactive molecules have been discovered in addition to the main nutrients, such as multipotent stem cells, hormones, immunoglobulins, and bacteria. Furthermore, the human milk oligosaccharides (HMOs) seem to exert several important protective biological functions. According to the HMOs’ composition, breast milk can be classified as a secretory or non-secretory phenotype. In our study, we investigated the metabolome of milk collected from 58 mothers that delivered neonates at term, that were appropriate, small or large for gestational age, by performing nuclear magnetic resonance spectroscopy (^1^H-NMR). From the data analysis, two groups were distinguished based on their different types of oligosaccharides, and classified according the mother phenotype: secretory and non-secretory. This information is of major importance given the different biological function of the different HMOs, such as immune-modulation and protection against disease. This would allow us to predict whether the neonate would be, for instance, more prone to developing certain diseases, and to tailor her or his nutrition to fit their needs perfectly and pave the way to a personalized nutrition.

## 1. Introduction

Maternal milk is a complex fluid that evolutionarily adapted to satisfy the nutritional needs of the neonate. In addition to the classical nutrients, such as proteins, carbohydrates, lipids, vitamins, and minerals, milk contains several bioactive components [1]. Among them there are growth factors, anti-microbial components, and stem cells, which are able to integrate in vivo in the tissues of the neonate and to differentiate in mature cells [2].

Human breast milk (HBM) has the characteristic of significantly varying from one woman to another, and it changes constantly during lactation to adapt to the growing energy needs of the developing infant. The variable components of HBM include oligosaccharides, which represent the third most abundant constituent. They exert several important functions, such as the modulation of the composition of the gut microbiota that influences a wide range of physiological processes in the neonate [3]. In fact, oligosaccharides are indigestible by the neonate, but they are a source of nourishment for the bacteria colonising and living in the neonate’s guts. Their presence can favor the growth of some species of commensal bacteria (i.e., *Bifidobacteria* strains) and inhibit the growth of pathogens. Furthermore, *Bifidobacteria spp*. can also exert direct antibacterial activity by modulating binding receptors, both on the pathogens and on the gut lumen blocking their binding and eventual damage. *Bifidobacteria spp.* also modify the cytokine response in T-cells. Moreover, since the types of oligosaccharides present in human milk is genetically determined, some profiles are thought to be more protective than others, even against respiratory tract infections. Finally, it is interesting to notice that according to a recent study, oligosaccharides are not altered by pasteurization or freeze-drying [4].

The application of metabolomics in neonatology offers an approach to investigate the complex relationships between nutrition and infant’s health. The characterization of the metabolome of HBM compared to formula milk allows understanding of how each nutrient affects the neonate’s metabolism, and offers the chance to intervene on the composition of the diet according to the nutritional request of the neonate [5,6,7,8,9].

In this study, we compared the metabolome of HBM samples collected from women that delivered neonates appropriately large or small for their gestational age and weight, in order to explore the presence of possible differences correlated to the fetal growth. The metabolomics analysis, performed by nuclear magnetic resonance spectroscopy (^1^H-NMR), provided information concerning the metabolic variability of the samples.

## 2. Results 

Table 1 shows the average characteristics of the enrolled mothers, grouped according to the fetal growth standards. Individual variables of each subject are reported in the Supplementary Materials (Table S1). No significant difference in age, body mass index (BMI), and gestational age (GA) were observed among groups. 

**Figure 1:**
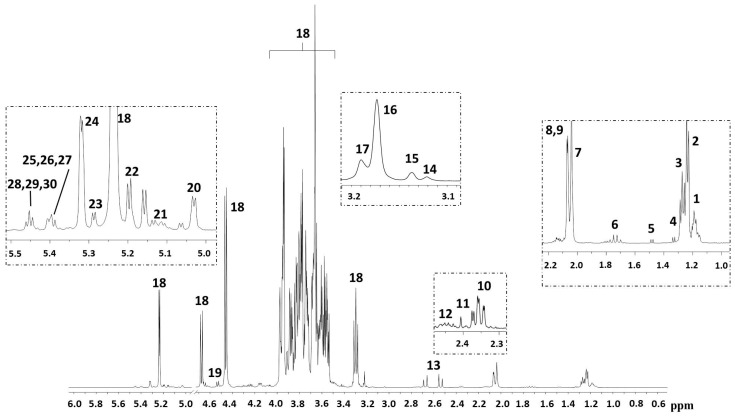
Representative nuclear magnetic resonance spectroscopy (^1^H-NMR) spectra of human breast milk. Chemical shift attribution was performed according to the literature [5,6,9,10]. Keys: (1) H-6 Fuc(α1-3)Glc in 3′ fucosyllactose, lacto-*N*-fucopentaose V, and lacto-*N*-difucohesaose II; H-6 Fuc(α1-3)GlcNAc in lacto-*N*-difucohesaose II; (2) H-6 Fuc(α1-2)Glc in 2′ fucosyllactose; H-3 Fuc(α1-2)Gal in 2′ fucosyllactose, and in lacto-*N*-fucopentaose; (3) H-6 Fuc(α1-2)Gal in lacto-*N*-difucohesaose I and in lacto-*N*-difucotetraose. (4) Threonine/lactate; (5) alanine; (6) 3′ sialyllactose/ 6′ sialyllactose; 7. *N*-Acetyl moieties; (8) CH3 GlcNAc(β1-6) in lacto-*N*-fucopentaose; (9) CH3 GlcNAc(β1-6) in lacto-*N*-difucohesaose I and branched; (10) glutamate; (11) succinate; (12) glutamine; (13) citrate; (14) choline; (15) O-phosphocholine; (16) glycerophosphocholine; (17) carnitine; (18) lactose; (19) H-1 Gal(β1-4); (20) H-1 Fuc(α1-4)GlcNAC in lacto-*N*-difucohesaose I-II; (21) lacto-*N*-fucopentaose III and branched; (22) H-1 Fuc(α1-2)Gal in lacto-*N*-difucohesaose I and branched; (23) H-1 Fuc(α1-2)Gal in lacto-*N*-difucotetraose; (24) H-1 Fuc(α1-2)Gal in 2′ fucosyllactose and in lacto-*N*-fucopentaose I, as well as branched; (25) H-1 Fuc(α1-3)αGlc in lacto-*N*-difucohesaose II; (26) H-1 Fuc(α1-3)αGlc in 3′ fucosyllactose and lacto-*N*-fucopentaose V; (27) H-1 Fuc(α1-3)αGlc in lacto-*N*-difucotetraose; (28) H-1 Fuc(α1-3)βGlc in lacto-*N*-difucohesaose II; (29) H-1 Fuc(α1-3)βGlc in 3′ fucosyllactose and lacto-*N*-fucopentaose V; (30) H-1 Fuc(α1-3)βGlc in lacto-*N*-difucotetraose.

**Figure 2:**
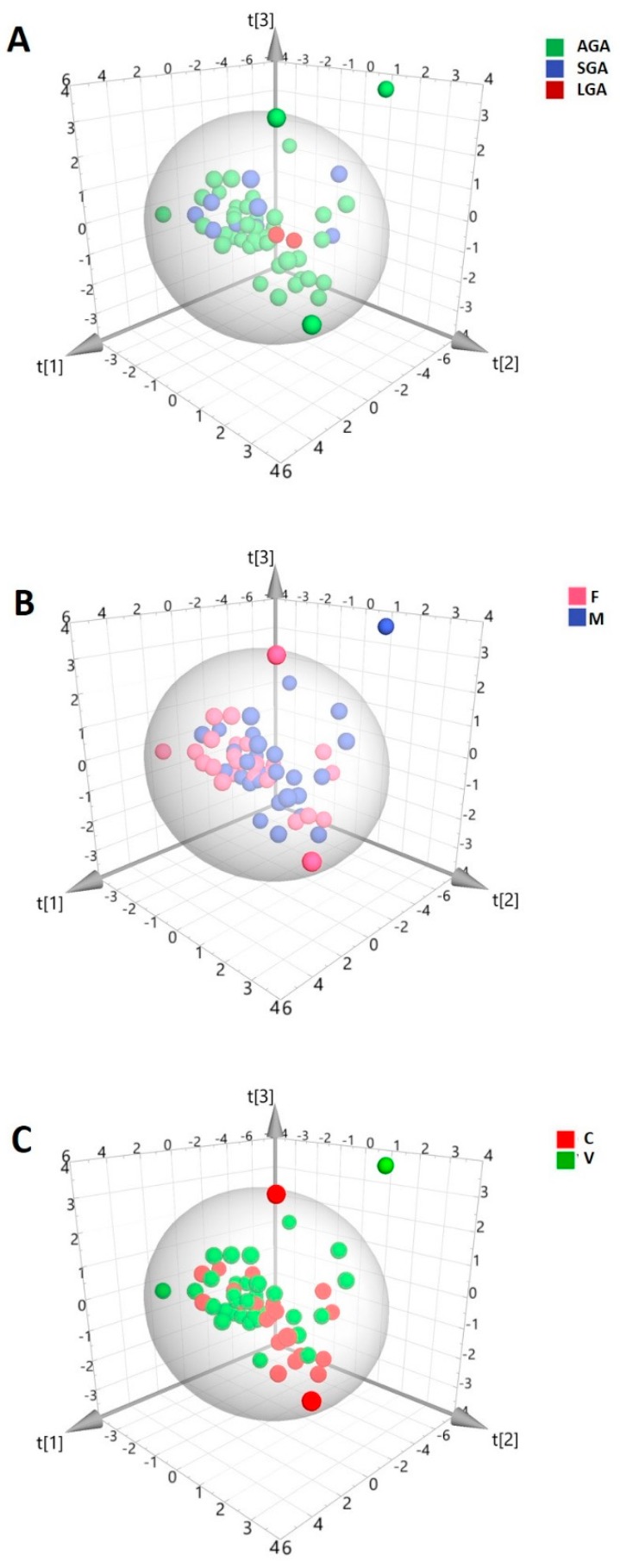
Three-dimensional (3D) principal components analysis (PCA) score plot of the overall data set of human breast milk (HBM) spectra from ^1^H-NMR. (PC1 = 37.8%; PC2 = 17.2%; PC3 = 13.1%). The scores are colored according to (**A**) neonatal customized centiles (AGA: appropriate for gestational age; LGA: large for gestational age; SGA: small for gestational age); (**B**) gender (F: female; M: male); (**C**) mode of delivery (C: cesarian; V: vaginal).

In total, 58 HBM samples were analyzed by ^1^H-NMR spectroscopy. A representative spectrum is shown in Figure 1. In order to discover intrinsic clusters and outliers within the data set, the NMR spectral data was analyzed with the unsupervised principal components analysis (PCA) approach. The 3D PCA scores plot explained 68.1% of the variation in the dataset (Figure 2). As can be seen, no clear separation of samples was observed in terms of neonatal customized centiles (Figure 2A), gender (Figure 2B), and mode of delivery (Figure 2C). For a further study of possible differences, with regard to the above-mentioned characteristics, supervised statistical methods were also applied, without achieving significant results (data not shown).

**Figure 3:**
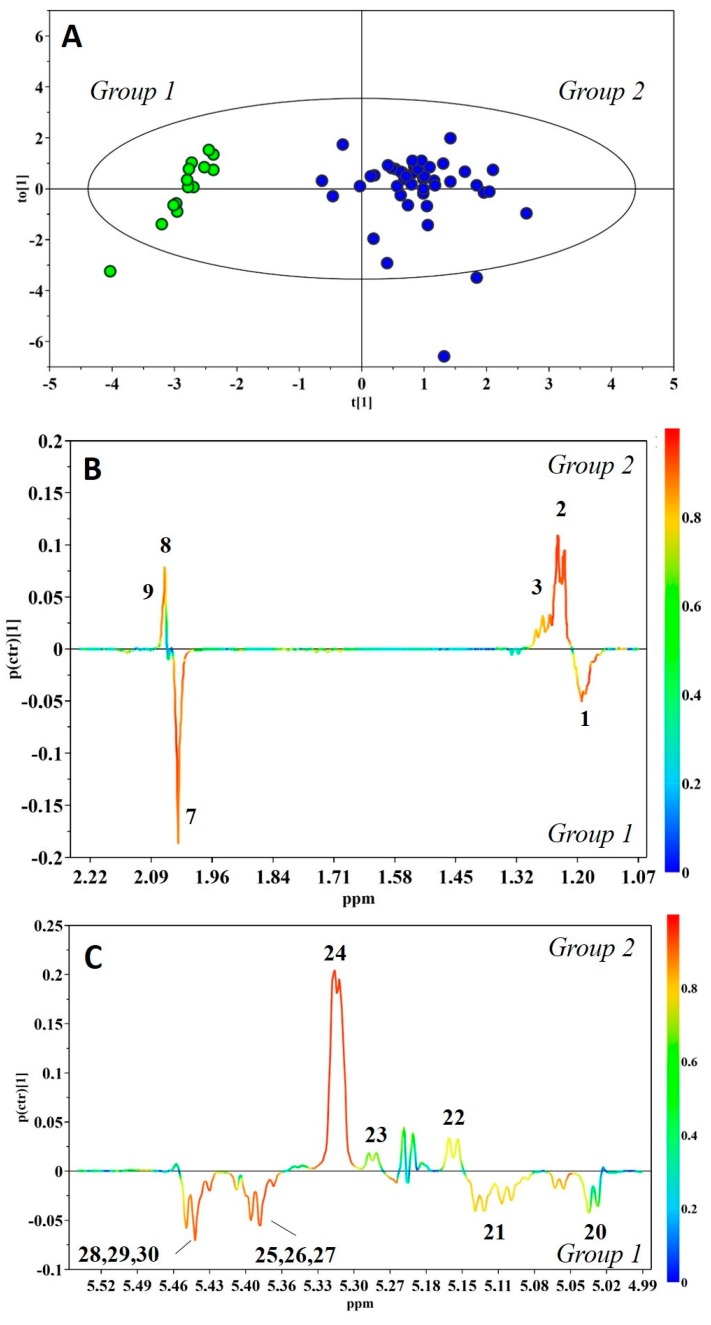
Orthogonal projection to latent structures discriminant analysis (OPLS-DA) score plot (**A**) and expansions of the loading plot of the human milk oligosaccharides’ (HMOs’) nuclear magnetic resonance (NMR) spectral regions (**B–C**) for the pair-wise comparison between Group 1 (green) and Group 2 (blue) (*R*^2^*Y* = 0.857, *Q*^2^*Y* = 0.809, *p* < 0.001). Peak codes are the same as Figure 1.

Nevertheless, exploring the three-dimensional (3D) PCA score distribution by hierarchical cluster analysis (HCA) demonstrated the presence of two main clusters, Group 1 and Group 2, containing 14 and 44 HBM samples, respectively (Figure S1 in Supplementary Materials). A subtle metabolic difference between the two groups was characterized by orthogonal projection to latent structures discriminant analysis (OPLS-DA), assigning the two clusters into classes. This allowed the construction of a very good model, which explained 85.7% and predicted 80.9% of the variation in Y (sample types), and explained 51.7% of the variation in X (NMR response variables). Figure 3 shows the corresponding scores and coefficient loading plots. The analysis of the loadings plot indicates that the most significant contribution of the variation between the two classes arose from human milk oligosaccharides (HMOs) (Figure 3B,C): The resonances arising from α1,3/4-linked fucosyloligosaccharide units, with fucose linked only by α1,3 or α1,4 glycosidic bonds, were strongly correlated with Group 1, while those arising from α1,2 fucosyloligosaccharide units were strongly correlated with Group 2. In particular, the visual inspection of the corresponding NMR spectra showed a lack of signals from α1,2 fucosyloligosaccharides in the milk of Group 1, and the presence of peaks from all fucosylated oligosaccharides in milk of Group 2. Based on the literature data [11], these differences are indicative of secretor status in lactating mothers. Thus, Group 1 was recognized to be composed of milk produced from non-secretor mothers, while Group 2 included milk samples from mothers with secretory phenotype. The OPLS-DA model was validated using the response of the permutation test (Figure S2 in Supplementary Materials), and additionally by an external validation approach, splitting the data set into calibration and test sets. Table 2 shows that the misclassification analysis was able to correctly classify all samples in the test set. 

## 3. Discussion

HMOs are associated with the same genes that determine Lewis blood type and secretor status. Fucosyltransferase 2 (FUT2) catalyses the addition of Fuc via α1–2 linkages on Lewis blood group epitopes, as well as on HMOs. FUT2 is actively expressed in over 70% of the population (secretors). Milk of secretor women contains high concentrations of α1–2-fucosylated HMOs. One of the dominant HMOs in the milk of secretor women is 2′-fucosyllactose (2′FL). Non-secretors, however, do not express an active FUT2, and the milk of non-secretor women lacks α1–2-fucosylated HMOs. In addition, 2′FL is almost completely absent. Fucosyltransferase 3 (FUT3), on the other hand, catalyses the addition of Fuc via an α1–3/4linkage (depending on the type of the underlying HMO backbone), and FUT3 can also be inactive in parts of the population (Lewis negative) [11]. Depending on the expression of active FUT2 and FUT3 enzymes, women can be separated into four groups: (1) Lewis-positive secretors (FUT2 and FUT3 active); (2) Lewis-negative secretors (FUT2 active, FUT3 inactive); (3) Lewis-positive non-secretors (FUT2 inactive, FUT3 active); and (4) Lewis-negative non-secretors (FUT2 and FUT3 inactive). 

Our data indicates a higher prevalence of the secretory phenotype in the study population under investigation, in accordance with Praticò et al. [6], Smilowitz et al. [7], and Spevacek et al. [8]. This finding is consistent with global incidence; in fact, according to epidemiological studies in France, Italy, Sweden, Germany, Mexico, and Japan, 80% of women display a secretory phenotype, while in Africa, Middle East, and Bangladesh, the percentage decreases to 50% [12]. However, the prevalence of the Lewis gene is 50% of the general population. 

The secretory phenotype can represent a tool to select genetic traits that better answer the environmental factors in the population. Indeed, maternal milk with the secretory phenotype is related to an enhanced protection against some pathogens, such as *E. Coli* and *Campylobacter*. This phenomenon can explain the prevalence of the secretory phenotype in specific regions where the pathogens are endemic. For instance, the low incidence of the non-secretory phenotype in the indigenous population of Mexico could be responsible for the higher vulnerability of neonates that receive milk containing few oligosaccharides that are α1,2-conjugated [13].

The set of discriminant metabolites that differentiate the phenotypes of milk oligosaccharides are 2′fucosyl-lactose (2′FL), lactodifucotetraose (LDFT), lacto-*N*-fucopentaose I (LNFP I), and lacto-*N*-difucoesaose I (LNFDH I), which reflect the maternal secretory state and are codified by the *FUT2* gene. Conversely, 3′fucosyl-lactose (3′FL) and lacto-*N*-difucoesaose III (LNFP III) are codified by the *FUT3* gene, and they are present in the non-secretory phenotypes that are Lewis-positive.

As already mentioned, according to literature, HMOs exert several positive effects on neonates’ health status. Indeed, lactofucoditetraose has anti-inflammatory properties, and suppresses platelet-induced inflammatory processes [14], while the lacto-*N*-fucopentaose is associated with decreased infant morbidity and improved growth rates [15]. In addition, 3′ fucosylactose seems to exert some protective effects against necrotizing enterocolitis (NEC), due to its preservation of vascular reactivity [16]. 

When it comes to the bacterial consumption of this particular type of saccharide, *Bifidobacterium longum* is one of the major consumers. This bacterial strain seems to be involved in improved response to some vaccines, and has some anti-inflammatory effects on the premature intestine. Thus, there is an indirect modulation of the immune system of the neonate host. Furthermore, it decreases the intestinal permeability, enhancing the protective mechanisms against NEC [17].

The characterization of the milk metabolome performed in the present study highlights a correlation between the phenotype expressed by the mother and the presence of specific oligosaccharides. However, no significant differences among samples were observed in terms of mode of delivery, gestational age, gender, and centile of the neonate. In line with the studies by Praticò et al. [6] and Smilowitz et al. [7], which show the presence of fucosylated oligosaccharides in position α1,2 (2′-FL, LDFT, LNFPI) in the secretory phenotype and oligosaccharides 3′-FL and LNFD II in the non-secretory phenotype, this study demonstrates that the milk samples of the non-secretory phenotype present 3′-fucosyl-lactose and other fucosylated oligosaccharides in the α1-3/4 position. In addition, the milk samples of the secretory phenotype present fucosylated oligosaccharides in the α1-2 position. In accordance with the study by Spevacek et al. [8], which showed the presence of fucosilated oligosaccharides, such as 3′FL, 2′FL, and LNFP III, our study also demonstrates the above-mentioned milk oligosaccharides; however, the variation in the first month of lactation was not documented.

In the future, it would be advisable to widen the range of cases and analyze the breast milk of preterm neonates in detail through metabolomics, since from the study performed so far it has been shown that being preterm presents a decrease in the fucosylation of HMOs. Preterm neonates are a population at risk, because of the immaturity of the immune system and other tissues, which exposes them to multiple infections—NEC in particular. Neonates fed with formula milk—or if their microbiota is dominated by *Proteobacteria* [18], or if the maternal milk does not contain fucosylated oligosaccharides—are at a higher risk of developing this pathology. Through the identification of preterm neonates of non-secretory mothers, a specific nutrition could be introduced to get the benefits of the α1–2 fucosylated oligosaccharides, and to establish a more strict infectious monitoring [19]. 

## 4. Material and Methods

### 4.1. Study Population

This study was approved by the Alexandra Hospital ethics committee in Athens. In total, 58 women admitted to the maternity ward of the Alexandra Hospital were recruited and divided into three groups, according to the fetal growth standard. By applying GROW (Gestation Related Optimal Weight computer generated program), we calculated the customized centiles for each newborn [20]. Thus, 48 newborns were appropriate for gestational age (AGA), 2 were large for gestational age (LGA), and 10 were small for gestational age (SGA). The characteristic of the study population are reported in Table 1. Breast milk (colostrum, 5–6 mL) was collected by nursing mothers, being in good general health and nutritional state on the third or fourth day postpartum, when milk production was sufficient. Breast milk was expressed by an electric breast pump. All samples were collected during the morning following breastfeeding. Samples were transferred within 20 min of sampling in the laboratory into special containers on ice. Centrifugation (20 min at 1500× *g*) in a refrigerated centrifuge (4 °C) took place immediately upon arrival. The milk’s undernatant layer was separated into two equal aliquots, which were immediately stored in a deep freezer (−80 °C) until metabolomic analysis.

### 4.2. Milk Sample Preparation

In order to remove residual lipids and proteins, 500 µL of milk samples were centrifuged at 10,000× *g* for 30 min at 4 °C, using Amicon Ultra 0.5 mL 10 kDa spin filters (Millipore, Billerica, MA, United States). Prior to filtration, filters were extensively washed with distilled water (500 µL per filter) to deprive the membrane of the embedded glycerol. The procedure was carried out iteratively until control by NMR spectroscopy of the wash water showed no residual presence of glycerol. Each filtered sample (350 μL) was mixed with 350 μL of 0.1 M phosphate buffer solution (pH 7.4) containing sodium 3-trimethylsilyl-(2,2,3,3-2H4)-1-propionate (TSP) (final concentration 2 mM), and then 650 µL were transferred into a 5 mm wide NMR tube.

### 4.3. Data Acquisition

The ^1^H NMR experiments were performed at 300 K on a Varian UNITY INOVA 500 spectrometer (Agilent Technologies, Inc., Santa Clara, CA, United States), operating at a frequency of 499.83 MHz. One-dimensional (1D) ^1^H NMR spectra were recorded using a standard pulse sequence (1D nuclear Overhauser effect spectroscopy), with pre-saturation during relaxation and mixing time for water suppression. Typically, 256 transients were acquired over a spectral width of 6000 Hz, with a total acquisition time of 1.5 s and a mixing time of 0.1 s. Prior to Fourier transformation, an exponential line-broadening factor of 0.3 Hz was applied to the free induction decays (FIDs). Then, spectra were phased and baseline-corrected, and the chemical shift scale was set by assigning a value of δ = 0.00 ppm to the signal for the internal standard TSP. 

### 4.4. Nuclear Magnetic Resonance Data Preparation

The NMR milk spectra were processed using MestReNova, version 8.1 (Mestrelab Research SL, Santiago de Compostela, Spain), and misalignments in the chemical shift mainly due to pH-dependent signals were corrected. Each spectrum was integrated (binned) using 0.001 ppm integral regions between 9.5 and 0.5 ppm, excluding the portions with the residual water (δ 4.6−5.2). Bins were normalized to the sum of the total spectral area, to compensate for the overall concentration differences. The final data set was reduced to ASCII files and converted into an Excel file.

### 4.5. Multivariate Analysis of Milk Metabolic Profiles

Data were imported into SIMCA 14 (MKS Data Analytics Solutions, Umea, Sweden) for statistical analysis. Principal component analysis (PCA) was performed to detect possible clusters or outliers. Hierarchical clustering analysis (HCA) was employed to explore the presence of similarities and dissimilarities among samples, by calculating and comparing the distances between pairs of scores in the 3D PCA space. Orthogonal projection to latent structures discriminant analysis (OPLS-DA) was applied to highlight significant biomarkers. The model quality was evaluated by (1) the goodness of fit, based on the proportion of variance in the *X* (*R*^2^*X*) and *Y* (*R*^2^*Y*) matrixes explained by the model; and (2) the goodness of prediction, based on the proportion of variance in the data that can be predicted by the model (*Q*^2^). The models were tested for overfitting by using *y*-table permutation testing (with 999 permutations) and a cross-validation tool [21] (the dataset was split into random subsets of training data and test data, used to build and validate the model, respectively). The significance of the models was further assed by an ANOVA based on the cross-validated predictive residuals (CV-ANOVA), with a significance threshold set to 0.05. 

## 5. Conclusions

The application of metabolomics to maternal milk is an emerging field that explores the relationship between metabolome and maternal diet, lifestyles, pathologies, and phenotype. Even if it is still in a developing stage, published studies show how metabolomics can be the key to improving knowledge about maternal milk complexities. 

The aim of this study was to compare the metabolome of HBM from mothers of AGA, LGA, and SGA neonates, collected on the third or fourth day postpartum. Our results did not point out any significant difference among the milk of the three groups of mothers when analyzed in terms of fetal growth (i.e., normal, too small, too large). Conversely, a significant separation of samples into two groups, independently of the classification of newborns by weight and gestational age, was observed based on the content of HMOs (secretory versus non-secretory phenotype). The important functions of the oligosaccharides are still being investigated, and thanks to the new analytical methodologies, it will be possible to know their role in metabolic pathways in humans.

The future of research concerning maternal milk seems to include metabolomics, microbiomics, and multipotent stem cells [22,23]. From the integration of these three fields, it probably will be possible to make further progress in the understanding of the composition and functions of maternal milk, which is of major importance for the health of the newborn. If it will be possible to know which types of human oligosaccharides are produced by a mother, the management of the breastfed neonate could change forever [24]. Indeed, if some oligosaccharides are missing, with the new synthesis technologies they could be integrated during feeding. Furthermore, thanks to this information, it could be possible to modulate infant microbiota, from a protective point of view—especially in preterm neonates, in which the overall immaturity of the organs make them more prone to infections and other diseases, such as NEC and bronchopulmonary dysplasia [25]. In the next five years, this class of nutrient could be the main actor of the tailored nutrition and care of the newborn [26].

## Figures and Tables

**Table 1:** Characteristics of the study population, according to fetal growth standards.

	AGA (*n* = 46)	SGA (*n* = 10)	LGA (*n* = 2)
Gestational age (weeks, mean ± SD)	39.3 ± 1.5	39.2 ± 1.7	38.6 ± 0.1
Maternal age (y, mean ± SD)	31.0 ± 5.4	31.4 ± 4.9	34.5 ± 3.5
Maternal BMI (kg/m^2^, mean ± SD)	24.4 ± 4.8	22.7 ± 4.3	20.2 ± 1.7
Cesarean delivery (%)	52	80	50
Gender (M/F)	25/21	3/7	2/0

AGA: Adequate for gestational age; SGA: small for gestational age; LGA: large for gestational age.

**Table 2:** Average misclassification table for validation of the OPLS-DA model.

Class	Members	Predicted	Corrected Predictions (%)
Group 1	5	5	100
Group 2	10	10	100

## Supplementary Materials

The following are available online at http://www.mdpi.com/2218-1989/8/4/79/s1, Figure S1: Dendogram of the hierarchical clustering analysis (HCA) performed on the 3D PCA model, Figure S2: Permutation test with 999 permutations of OPLS-DA model: *R*^2^*Y* intercept = 0.113; *Q*^2^*Y* intercept = −0.348, Table S1: Characteristics of study population.
