# Supplementary material for: Metabolomics of Breast Milk: The Importance of Phenotypes
Source: Metabolites. 2018 Nov 20;8(4):79. doi: 10.3390/metabo8040079 (PMC6315662; doi:10.3390/metabo8040079)
Supplement: Supplementary file 1 [file metabolites-08-00079-s001.pdf]

# SUPPORTING INFORMATION

## Metabolomics of breast milk: the importance of phenotypes

Angelica Dessì[1§] *, Despira Briana[2§] , Sara Corbu[1], Stavroula Gavrili[3], Flaminia Cesare Marincola[4], Sofia Georgantzi[3], Roberta Pintus[1], Vassilios Fanos[1], Ariadne Malamitsi-Puchner[2]

1 Neonatal Intensive Care Unit, Neonatal Pathology and Neonatal Section, Azienda Ospedaliera Universitaria, University of Cagliari, Monserrato, Italy

2 1st Department of Paediatrics, "Aghia Sophia" Children's Hospital, National and Kapodistrian University of Athens, Athens, Greece.

3 Neonatal Intensive Care Units, General District, Hospital Alexandra, Athens, Greece.

4 Department of Chemical and Geological Sciences, University of Cagliari, Monserrato, CA, Italy

* Corresponding author: angelicadessi@unica.it

[§] The authors contributes equally to the work

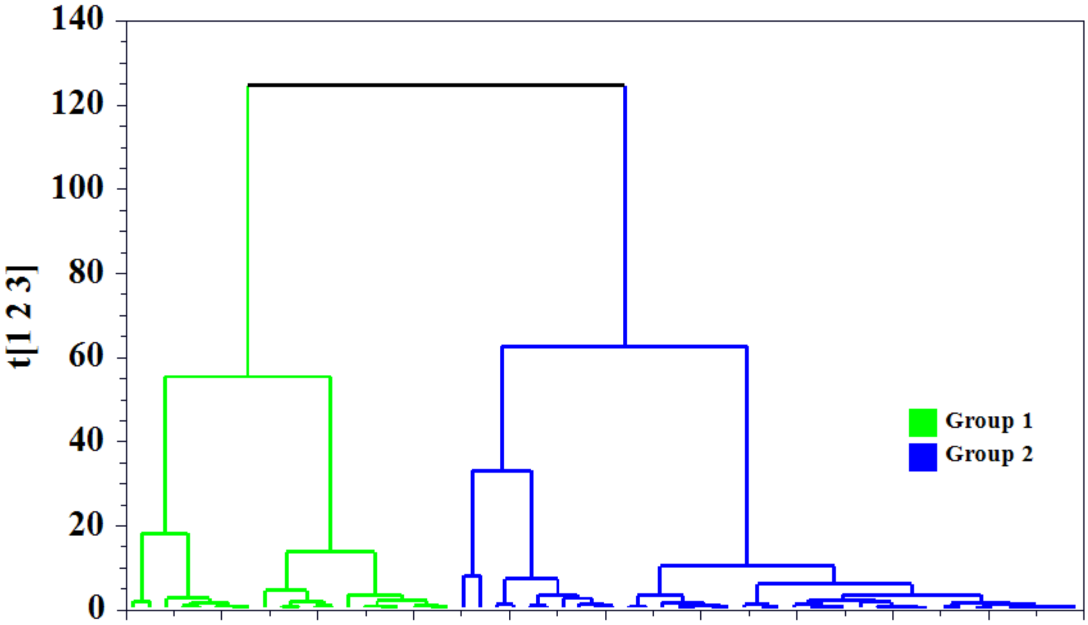


**Figure S1.** Dendogram of the hierarchical clustering analysis (HCA) performed on the 3D PCA model

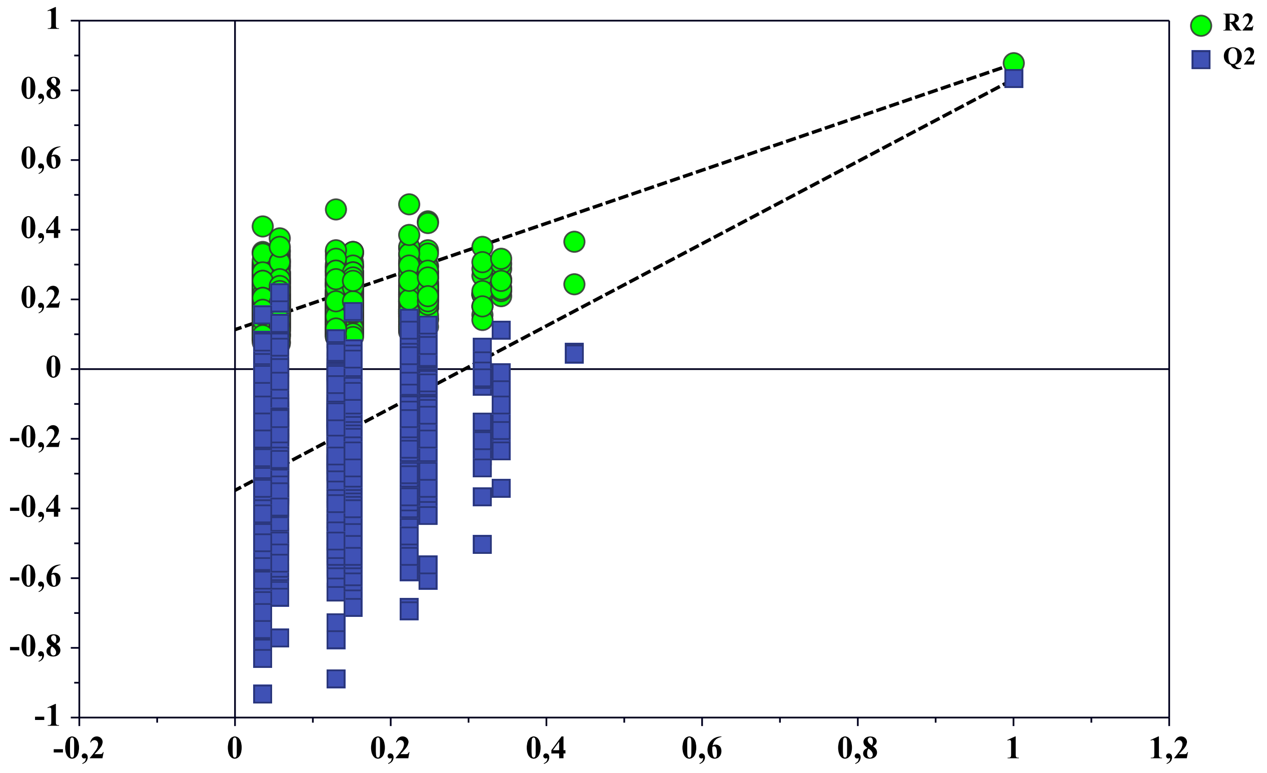


**Figure S2.** Permutation test with 999 permutations of OPLS-DA model: $R^2Y$ intercept = 0,113; $Q^2Y$ intercept = -0,348.

**Table 1.** Characteristics of study population

| Sample code | Mother | | | | | Newborn | |
|---|---|---|---|---|---|---|---|
| | Age (years) | BMI ($Kg/m^2$) | Milk phenotype[1] | Gestational age (weeks+days) | Mode of delivery | Gender | Centile |
| *AGA* | | | | | | | |
| **HBM1** | 19 | 15.8 | *Secretor* | 37+1 | C | F | 20 |
| **HBM2** | 20 | 19.7 | *Secretor* | 39+1 | V | M | 15 |
| **HBM3** | 21 | 26.4 | *Secretor* | 40+1 | V | M | 15 |
| **HBM4** | 21 | 18.6 | *Secretor* | 39 | V | F | 20 |
| **HBM5** | 24 | 26.8 | *Secretor* | 35+3 | C | F | 27 |
| **HBM6** | 24 | 33 | *Secretor* | 39+1 | C | F | 40 |
| **HBM7** | 26 | 22.2 | *Secretor* | 39+4 | V | M | 37 |
| **HBM8** | 26 | 33.2 | *Non secretor* | 39+2 | V | F | 40 |
| **HBM9** | 26 | 21.3 | *Non secretor* | 39+1 | V | F | 52 |
| **HBM10** | 26 | 29.6 | *Non secretor* | 38+6 | C | F | 72 |
| **HBM11** | 27 | 25.4 | *Secretor* | 39+5 | V | M | 35 |
| **HBM12** | 27 | 33 | *Secretor* | 38+5 | V | M | 42 |
| **HBM13** | 27 | 21.1 | *Secretor* | 39+5 | C | M | 59 |
| **HBM14** | 28 | 20 | *Secretor* | 39+1 | V | M | 26 |
| **HBM15** | 28 | 21 | *Secretor* | 39+1 | V | M | 56 |
| **HBM16** | 29 | 23 | *Secretor* | 40 | V | F | 18 |
| **HBM17** | 29 | 25 | *Secretor* | 40+5 | C | F | 67 |
| **HBM18** | 29 | 20.4 | *Secretor* | 40+1 | V | F | 75 |
| **HBM19** | 30 | 20.4 | *Secretor* | 38+3 | C | M | 44 |
| **HBM20** | 30 | 20 | *Secretor* | 40+3 | C | M | 45 |
| **HBM21** | 31 | 24.1 | *Secretor* | 38+6 | C | M | 40 |
| **HBM22** | 32 | 26 | *Secretor* | 37 | V | F | 21 |
| **HBM23** | 32 | 22 | *Secretor* | 38+5 | C | M | 33 |
| **HBM24** | 32 | 20 | *Non secretor* | 38+6 | C | M | 39 |
| **HBM25** | 32 | 22 | *Non secretor* | 40 | V | F | 57 |
| **HBM26** | 32 | 32 | *Secretor* | 38+6 | V | M | 75 |
| **HBM27** | 33 | 21.5 | *Secretor* | 40+1 | C | M | 44 |
| **HBM28** | 33 | 23.4 | *Secretor* | 38+6 | C | M | 54 |
| **HBM29** | 34 | 33 | *Secretor* | 40 | V | F | 20 |
| **HBM30** | 34 | 25.9 | *Secretor* | 38+4 | V | M | 40 |

| | | | | | | | |
|---|---|---|---|---|---|---|---|
| **HBM31** | 34 | 19.3 | *Non secretor* | 38+6 | C | M | 43 |
| **HBM32** | 34 | 26 | *Secretor* | 38 | C | F | 72 |
| **HBM33** | 35 | 23.1 | *Secretor* | 38+3 | C | F | 44 |
| **HBM34** | 36 | 32 | *Secretor* | 39+6 | C | F | 17 |
| **HBM35** | 36 | 21.4 | *Secretor* | 40+1 | V | M | 30 |
| **HBM36** | 36 | 22 | *Secretor* | 38+5 | C | M | 32 |
| **HBM37** | 36 | 20.6 | *Secretor* | 39+2 | C | M | 45 |
| **HBM38** | 36 | 20.9 | *Secretor* | 38+6 | V | M | 65 |
| **HBM39** | 36 | 30 | *Secretor* | 39+5 | V | M | 69 |
| **HBM40** | 36 | 22.2 | *Non secretor* | 38+6 | C | F | 75 |
| **HBM41** | 37 | 30.8 | *Secretor* | 40+2 | V | F | 49 |
| **HBM42** | 37 | 21 | *Non secretor* | 36+1 | C | F | 50 |
| **HBM43** | 38 | 23.2 | *Secretor* | 41 | V | M | 23 |
| **HBM44** | 39 | 35.4 | *Secretor* | 40 | V | M | 25 |
| **HBM45** | 39 | 26.9 | *Secretor* | 38+6 | C | F | 40 |
| **HBM46** | 42 | 21.4 | *Non secretor* | 40 | V | F | 70 |
| ***SGA*** | | | | | | | |
| **HBM47** | 23 | 21 | *Secretor* | 37+1 | C | F | 5 |
| **HBM48** | 25 | 34 | *Non secretor* | 40 | V | M | 2 |
| **HBM49** | 26 | 21 | *Secretor* | 39 | V | F | 4 |
| **HBM50** | 32 | 22.2 | *Secretor* | 38+4 | V | F | 1 |
| **HBM51** | 33 | 19.6 | *Non secretor* | 39+1 | V | F | 1 |
| **HBM52** | 33 | 23.6 | *Secretor* | 38+5 | V | M | 7 |
| **HBM53** | 34 | 23.4 | *Non secretor* | 39 | V | M | 4 |
| **HBM54** | 36 | 20 | *Non secretor* | 39+6 | V | F | 5 |
| **HBM55** | 36 | 23.6 | *Secretor* | 39+2 | C | F | 9 |
| **HBM56** | 36 | 18.5 | *Non secretor* | 39 | V | F | 10 |
| ***LGA*** | | | | | | | |
| **HBM57** | 37 | 19 | *Secretor* | 38+5 | C | M | 92 |
| **HBM58** | 32 | 21.4 | *Secretor* | 39 | V | M | 91 |

**Abbreviations:** AGA: adeguate for gestational age; SGA: small for gestational age; LGA: large for gestational age; BMI: body mass index; C: cesarean section; V: vaginal delivery; F: female; M: male.

[1]Milk phenotype established according to NMR spectra analysis.
